# The circular RNA hsa_circ_000780 as a potential molecular diagnostic target for gastric cancer

**DOI:** 10.1186/s12920-021-01096-6

**Published:** 2021-11-27

**Authors:** Jian Song, Shuyong Yu, Dunjing Zhong, Weizhong Yang, Zhen Jia, Guihong Yuan, Ping Li, Ronglin Zhang, Yini Li, Guobing Zhong, Zhaowei Chen

**Affiliations:** 1 Department of Gastroenterology, The Affiliated Cancer Hospital of Hainan Medical University, Haikou, 570123 China; 2 Department of Gastrointestinal Surgery, The Affiliated Cancer Hospital of Hainan Medical University, Haikou, 570123 China; 3 Department of Digestive Endoscopy, The Affiliated Second Hospital of Hainan Medical University, Haikou, 570100 China; 4 Department of Anesthesiology, The Affiliated Cancer Hospital of Hainan Medical University, Haikou, 570123 China; 5 Department of Clinical Laboratory, The Affiliated Cancer Hospital of Hainan Medical University, Haikou, 570123 China

**Keywords:** Circular RNA, Gastric cancer, hsa_circ_000780

## Abstract

**Background:**

The present study aimed to identify a specific circular RNA (circRNA) for early diagnosis of gastric cancer (GC).

**Methods:**

Totally 82 patients with GC, 30 with chronic nonatrophic gastritis and 30 with chronic atrophic gastritis were included in this study. Four of the 82 GC patients were selected for screening. Total RNA from malignant and adjacent tissue samples was extracted, and circRNAs in four patients were screened. According to the screening results, the eight most upregulated and downregulated circRNAs with a statistically significant association with GC were identified by real-time fluorescent quantitative polymerase chain reaction (PCR). Then, the most regulated circRNA was selected for further sensitivity and specificity assessments. CircRNA expression was examined by quantitative reverse transcriptase PCR in 78 GC (21 and 57 early and advanced GC, respectively) and adjacent tissue samples, as well as in gastric fluid samples from 30 patients with chronic nonatrophic gastritis, 30 with chronic atrophic gastritis, and 78 GC.

**Results:**

A total of 445 circRNAs, including 69 upregulated and 376 downregulated circRNAs, showed significantly altered expression in GC tissue samples. Hsa_circ_000780 was significantly downregulated in 80.77% of GC tissue samples, with levels in GC tissue samples correlating with tumor size, tumor stage, T stage, venous invasion, carcinoembryonic antigen amounts, and carbohydrate antigen 19–9 levels. Strikingly, this circRNA was found in the gastric fluid of patients with early and advanced GC.

**Conclusions:**

The present study uncovered a new circRNA expression profile in human GC, with hsa_circ_000780 significantly downregulated in GC tissue and gastric fluid specimens. These findings indicate that hsa_circ_000780 should be considered a novel biomarker for early GC screening.

## Background

Gastric cancer (GC) ranks third in global cancer mortality, and is the most common cause of cancer deaths in China [1]. As GC is difficult to diagnose in the early stage, it is crucial to develop a noninvasive molecular diagnostic tool for GC detection [2]. Presently, the gold standard for early diagnosis of GC is gastroscopy. However, China has a large population, with inadequate awareness of cancer prevention and low compliance of gastroscopy screening; in addition, the number of digestive endoscopists cannot meet the needs of the general population for gastroscopy screening [3]. In recent years, robust advances in human genome sequencing, epigenetics, circular RNA (circRNA) assessment tools, and other molecular biological techniques have enabled the search for molecular diagnostic targets for GC. Gene molecular targets are widely distributed in the human body (blood, urine, feces, and various body fluids); additionally, the samples are easily obtainable, and the detection technology is mature. Among the various methods for studying gene mutations, circRNAs are a promising target for the molecular diagnosis of GC [4–9].

CircRNAs are closed circular genetic structures with no 3′-end poly-A structure and 5′-an end cap structure [10]. They range from hundreds to thousands of base pairs in length, and are not degraded by RNA exonuclease; circRNAs are stable in nature and exist widely in the biological community, with evolutionary conservatism [11]. Studies have reported that while circRNAs are widely considered miRNA sponges [12], not many of them own more predicted miR-binding sites than expected [13, 14]. Recent studies have shown abnormal circRNA expression in various tumor cells affects tumor occurrence, proliferation, and invasion [15–17]. Due to the stability of circular RNAs, they have been increasing investigated as potential tumor markers in recent years [18–20], especially in GC [21]. Scientist have observed that circ_002059, circ_0000745, circ_00000181, circ_0047905, circ_0014717, circ_0001017, and circ_0061276 are significantly downregulated in patients with GC, with good sensitivity and specificity in the diagnosis of GC [21–26]. However, no report has assessed circ_000780. Additionally, the role of microRNAs has been highlighted in the development and maintenance of drug resistance in GC, which is the most critical cause of GC treatment failure. CircRNAs act as miRNA sponges and affect gene regulation and expression [27, 28]. Although the global circRNA expression profile in human GC continues to be investigated, no circRNA with a clinical value in GC has been reported. Moreover, the role of circRNAs in early diagnosis of GC is not fully understood. Therefore, the present study aimed to identify a specific circRNA for early diagnosis of GC.

## Methods

### Sample collection

A total of 82 patients with GC admitted to the Cancer Hospital Affiliated to Hainan Medical College and examined in the endoscopy center from January 2017 to December 2018 were recruited in this study after institutional ethics clearance. Inclusion criterial were: (1) < 80 years of age; (2) complete clinical data available; (3) scheduled selective GC surgery; (4) no previous chemotherapy besides adjuvant treatment before operation; (5) no active gastrointestinal bleeding or obstruction. Exclusion criteria were: (1) uncontrolled diabetes or hypertension, coronary heart disease, stroke, cardiovascular, and/or cerebrovascular diseases; (2) severe underlying diseases such as pulmonary, liver, and/or kidney dysfunctions; (3) requiring resection of other organs. Of the 82 patients, four were selected for the circRNA chip screening study. They included two men (one with T3N1M0, moderately differentiated adenocarcinoma; one with T3N2M0, poorly differentiated adenocarcinoma) and two women (one with T3N1M0, moderately differentiated adenocarcinoma; one with T3N2M0, poorly differentiated adenocarcinoma). The average age, weight, and height of the four patients were 56.7 years, 58.3 kg, and 168 cm, respectively. The remaining 78 patients with GC (Table 1) were selected for endoscopic biopsy and gastric fluid sample collection. These patients were included in the validation study of differential circRNA expression. The diagnostic criteria for early GC (EGC) and advanced GC (AGC) were based on the National Comprehensive Cancer Network clinical practice guidelines in oncology (version 3.2016). Additionally, 30 patients with chronic nonatrophic gastritis (CNAG) and 30 with chronic atrophic gastritis (CAG) were randomly selected as the control group. The diagnostic criteria for CNAG and CAG were according to the consensus opinion of the 2012 Chinese Chronic Gastritis of Gastroenterology Branch of the Chinese Medical Association.

**Table 1:** Patient features

Characteristic	CNAG (*n* = 30)	CAG (*n* = 30)	EGC (*n* = 21)	AGC (*n* = 57)
Male	15	15	11	29
Female	15	15	10	28
Age (years)	51.7 ± 9.6	58.9 ± 9.5	59.6 ± 10.3	58.8 ± 8.6
BMI (kg/m^2^)	25.5 ± 2.5	24.8 ± 1.8	25.8 ± 2.3	23.3 ± 1.6
CEA (ng/ml)	–	–	4.56 ± 1.5	35.7 ± 3.5
CA199 (u/ml)	–	–	25.7 ± 3.5	78.6 ± 9.5

CNAG, chronic nonatrophic gastritis, CAG, chronic atrophic gastritis, EGC, early gastric cancer, AGC, advanced gastric cancer

GC specimens were obtained by cutting 0.5 cm^3^ of the whole layer of the GC tissue, whereas paracancerous tissue specimens were obtained by cutting 0.5 cm^3^ of the mucosa at least 5 cm away from the tumor body. The samples were separated from the body, quickly sliced to the required size, placed into storage tubes and stored in liquid nitrogen.

Endoscopic tissue and gastric juice samples were extracted from 78 patients with GC (21 patients with EGC and 57 with AGC), 30 with CNAG, and 30 with CAG. Table 1 illustrates the baseline characteristics of the patient and control groups. All specimens were collected and pretreated according to a previously described protocol and preserved at − 80 °C until RNA extraction [29].

### Total RNA extraction and reverse transcription

Total RNA from tissue and gastric fluid samples were extracted using TRIzol reagent (Invitrogen, Life Technologies Inc., Germany). RNA concentration was measured by reading absorbance at 260 nm (OD_260_) on a NanoDrop ND-1000 instrument (Thermo Fisher Scientific, DE, USA). RNA integrity was verified by denaturing agarose gel electrophoresis. Finally, total RNA was transcribed into cDNA through the GoScript Reverse Transcription (RT) system (Promega, WI, USA) following the manufacturer’s protocol.

### Microarray hybridization of circRNAs

GC tissue samples and matched adjacent noncancerous tissue specimens were selected for circRNA expression profiling using Human circRNA Array v2 (Arraystar, MD, USA). Total RNA was digested with RNase R (20 U/μL, Epicentre, Inc., Madison, WI, USA) to remove linear RNAs and enrich circRNAs. The enriched circRNAs were amplified and transcribed into fluorescent cRNA by the random priming method (Super RNA Labelling Kit; Arraystar). Labeled cRNAs were hybridized onto Human circRNA Array v2 (8 × 15 K, Arraystar). Slides were incubated for 17 h at 65 °C in a hybridization oven (Agilent, CA, USA). After washing the slides, the arrays were scanned on an Agilent Scanner (G2505C). The scanned images were then imported into the Agilent Feature Extraction software for grid alignment and data extraction. Quantile normalization and subsequent data processing were performed with the R software package. The expression profile of circRNAs, identified through volcano plot filtering between GC and paired adjacent noncancerous tissue samples, was statistically significant [fold change (FC) ≥ 2.0 and *P* ≤ 0.05]. Hierarchical clustering was performed to depict the distinguishable expression pattern of circRNAs among samples. The circRNA/microRNA interaction was predicted using TargetScan [30] & miRanda [31].

### Quantitative reverse transcription–polymerase chain reaction

The eight most upregulated and downregulated circRNAs exhibiting the greatest differences in expression between groups were selected for quantitative reverse transcription–polymerase chain reaction (qRT–PCR) verification in the four GC specimens and their adjacent tissues. qRT–PCR was performed with GoTaq qPCR Master Mix (Promega) on an Mx3005P Real-Time PCR System (Stratagene, CA, USA) in accordance with the manufacturer’s protocols. Divergent primers of the top eight upregulated and downregulated circRNAs and convergent primers of β-actin (H) were designed and synthesized by Aksomics (Shanghai) Biotechnology Co. Ltd. The divergent primers could only amplify circRNA and differentiate contaminants from the linear isoforms. Table 2 lists the circRNA primer sequences used for this procedure.

**Table 2:** Primer sequences for the assessed circRNAs

Gene	Primers
β-actin (H)	F: 5′-GTGGCCGAGGACTTTGATTG-3′ R: 5′-CCTGTAACAACGCATCTCATATT-3′
Hsa_circ_000102	F: 5′-AACGTATGAGGGTAGAAGAGAGA-3′ R: 5′-TCAGGTCTATAATCAATTTCATCTC-3′
Hsa_circ_000320	F: 5′-AATCTTAAGGGGCCAAAATTG-3′ R: 5′-TCCATTTTGGGTCCTTTGATT-3′
Hsa_circ_000324	F: 5′-GTAAGTAAGTGCCCGCACCATA-3′ R: 5′-CAGCGTGTTAGCAACAGAACC-3′
Hsa_circ_000780	F: 5′-TAGGAAACCTGCTGTGGAGTG-3′ R: 5′-AAGGGAACTATACAAGGAAATGC-3′
Hsa_circ_007738	F: 5′-ACATTGAGGAAGAAGGGCAGTA-3′ R: 5′-TTCAAGAGGGCTTACCTGGTA-3′
Hsa_circ_047478	F: 5′-CAGGAAGCCTAAAGGATTTTG-3′ R: 5′-CTTCAATAAACGGGAGGTGGT-3′
Hsa_circ_049637	F: 5′-ATTAAATTTTGTGTCTCCGCG-3′ R: 5′-CCTTTAAAACGACCCTCCG-3′
Hsa_circ_102411	F: 5′-CACCAACGACCATGAGAAGGTG-3′ R: 5′-AAGGACAGCAGGACGCAGAC-3′
Hsa_circ_103128	F: 5′-CAAGCACCAAAAGCAAGAAA-3′ R: 5′-CAGCGGCAAAACTATAACACC-3′
Hsa_circ_104293	F: 5′-GCACAGATCTGATTCTGAACGT-3′ R: 5′-TCATTGGATATGTCCTGATAGTCC-3′
Hsa_circ_404798	F: 5′-CTTCCGAATGCAAGAAAGATTG-3′ R: 5′-CCCTTACGGTACTTGAGGACC-3′
Hsa_circ_000250	F: 5′-GGGAGTGGCTGTGGATAAGT-3′ R: 5′-AGCATTTTTGTGAAATGGTGC-3′
Hsa_circ_018497	F: 5′-GTGATGGATATGATGGTGCAT-3′ R: 5′-AGTCCACGAAGTCGTACTGTC-3′
Hsa_circ_008882	F: 5′-TGGCAGCCTAGCATTAGC-3′ R: 5′-AGGGAGGTTGAAGTGAGAGGT-3′
Hsa_circ_002699	F: 5′-TGAGCACTGCTTTAATAGGGG-3′ R: 5′-GCCTTCATTATGAGAGGTTTATC-3'

RT-PCR was performed as follows: 40 cycles of 95 °C for 10 s and 60 °C for 60 s for amplification; annealing at 95 °C for 10 s, 60 °C for 60 s, and 95 °C for 15 s with slow heating from 60 to 99 °C (at 0.05 °C/s).

The target and housekeeping genes in each sample were analyzed by RT-PCR. According to the gradient dilution DNA standard curve, the expression levels of the target and housekeeping genes in each sample were directly generated on an Applied Biosystems ViiA™ 7 Real-Time PCR System (ThermoFisher Scientific, USA). Target gene concentration in each sample divided by that of the housekeeping gene was considered the relative expression level of the gene.

### Statistical analysis

Statistical analyses were performed with the SPSS 22.0 software (SPSS, IL, USA). When comparing the GC and paired noncancerous tissue groups for profile differences, the “FC” (ratio of group averages) between the groups for each circRNA was computed. The statistical significance of the difference was estimated by the *t* test. CircRNAs with FCs ≥ 2.0 were considered to be significantly differentially expressed. The analysis outputs were filtered, and differentially expressed circRNAs were ranked according to characteristics such as FC value, *P* value, and chromosome location. Differences in hsa_circ_000780 levels between the GC and paired adjacent noncancerous tissues were assessed by the *t* test for paired data; multiple groups (CNAG, CAG, EGC, and AGC) were assessed by one-way analysis of variance with post-hoc LSD test. Correlations between hsa_circ_000780 levels and clinicopathological factors were further analyzed by the Analyze–Correlate–Bivariate menu of SPSS 22.0. A *P* value < 0.05 was considered statistically significant.

## Results

### Profiles of circRNAs in GC

**Fig. 1:**
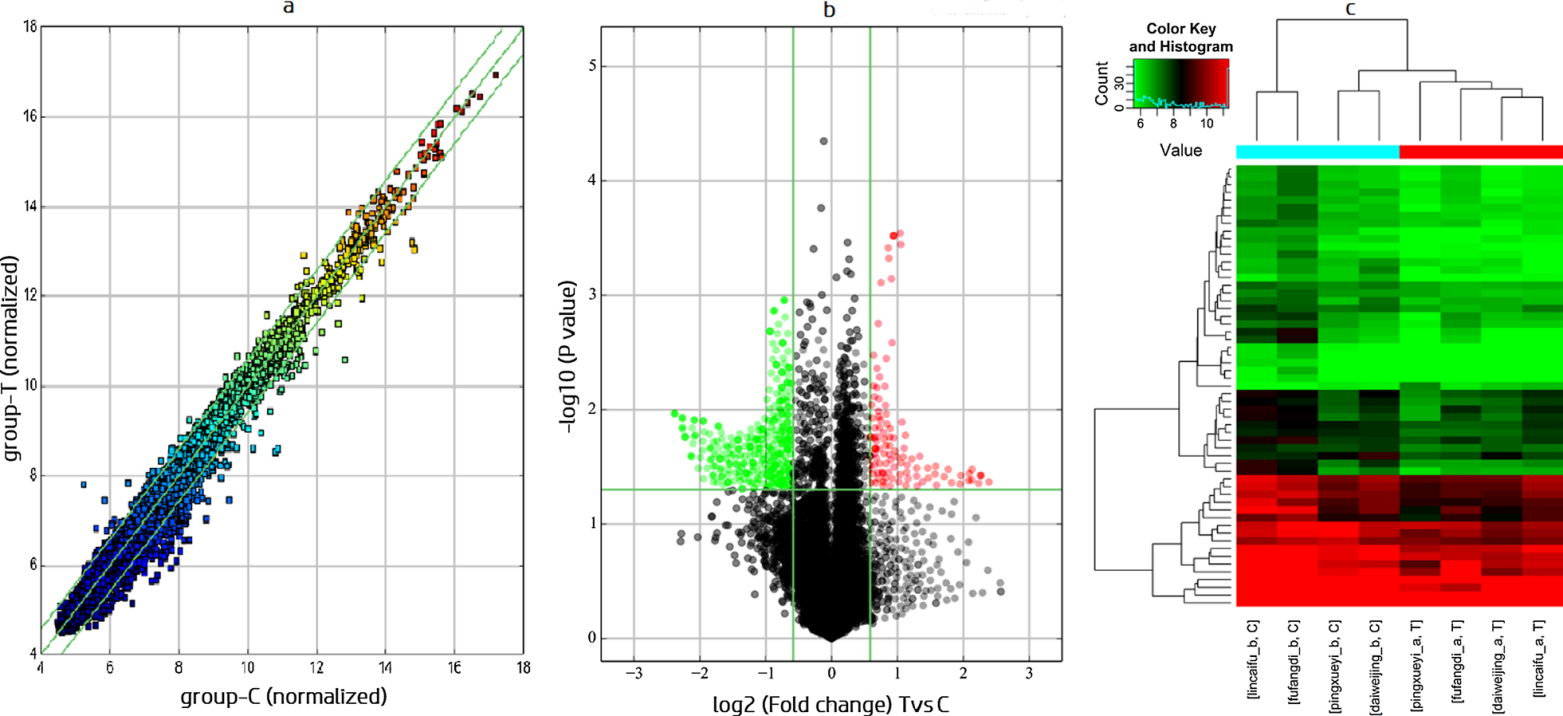
The circRNA expression profiles in GC and paired adjacent noncancerous tissues. **a** Scatter plots were used to compare circRNA expression levels between GC and paired adjacent noncancerous tissues. **b** Volcano plots were used to visualize the differential expression of circRNAs between GC and paired adjacent noncancerous tissues. The red and green points in the plot represent the differentially expressed circRNAs with statistical significance. **c** Hierarchical cluster analysis of circRNAs expressed in GC (red bar) and paired adjacent noncancerous (blue bars) samples

A total of 13,617 circRNAs were detected in the assessed GC and paired noncancerous samples by circRNA microarray analysis. Among them, 445 circRNAs were aberrantly expressed with statistical significance (P < 0.05 and FC > 2.0) between the GC and paired noncancerous tissues. FC filtering (Fig. 1a) or volcano plot filtering (Fig. 1b) was used to identify circRNAs whose differential expression was statistically significant. Hierarchical clustering was performed to depict the differential circRNA expression pattern among samples (Fig. 1c). Of the 445 circRNAs, 69 (15.51%) were significantly upregulated and 376 (84.49%) were significantly downregulated. The eight most upregulated and downregulated circRNAs, respectively, which were screened out and then validated in 4 pairs of gastric cancer and adjacent tissue samples are listed in Table 3. The expression of hsa_circ_000780 was the most altered in cancer tissue samples versus adjacent tissue specimens (*P* = 0.001240).

**Table 3:** The eight most upregulated and eight most downregulated circRNAs in GC

CircRNA ID	Chromosome	Regulation	Fold change	Strand	Gene Symbol	*P* value (Screening)	*P* value (qRT-PCR)
Has_circ_000780	chr10	Down	2.3500864	–	FAM107B	0.0212	0.0012
Has_circ_047478	chr18	Up	2.5070284	+	KIAA1328	0.0174	0.0204
Has_circ_404798	chr10	Down	2.6790388	–	DNMBP	0.0185	0.0251
Has_circ_104293	chr7	Up	2.0857679	–	FBXL18	0.0219	0.0376
Has_circ_008882	chrM	Down	6.0137146	+	MTND5	0.0120	0.18830
Has_circ_000102	chr1	Up	2.4795408	–	AKNAD1	0.0398	0.2428
Has_circ_049637	chr19	Down	4.6708359	+	CALR	0.0173	0.2519
Has_circ_103128	chr21	Down	2.5589795	+	DYRK1A	0.0492	0.412
Has_circ_405324	chr15	Down	3.2099669	+	STARD9	0.0261	0.4207
Has_circ_102411	chr19	Down	2.7435361	–	MFSD12	0.0247	0.4911
Has_circ_000250	chr18	Up	2.2513738	–	SMAD7	0.0368	0.6828
Has_circ_000320	chr11	Down	2.4551818	–	AHNAK	0.0359	0.7703
Has_circ_000324	chr11	Up	2.0957677	+	NEAT1	0.0452	0.8573
Has_circ_018497	chr10	Up	2.0093889	+	HNRNPH3	0.0467	0.8615
Has_circ_007738	chr9	Up	2.9836255	–	SHC3	0.0374	0.8718
Has_circ_002699	chr7	Up	2.7422086	+	MET	0.0266	0.8893

### Expression of hsa_circ_000780 in GC

**Fig. 2:**
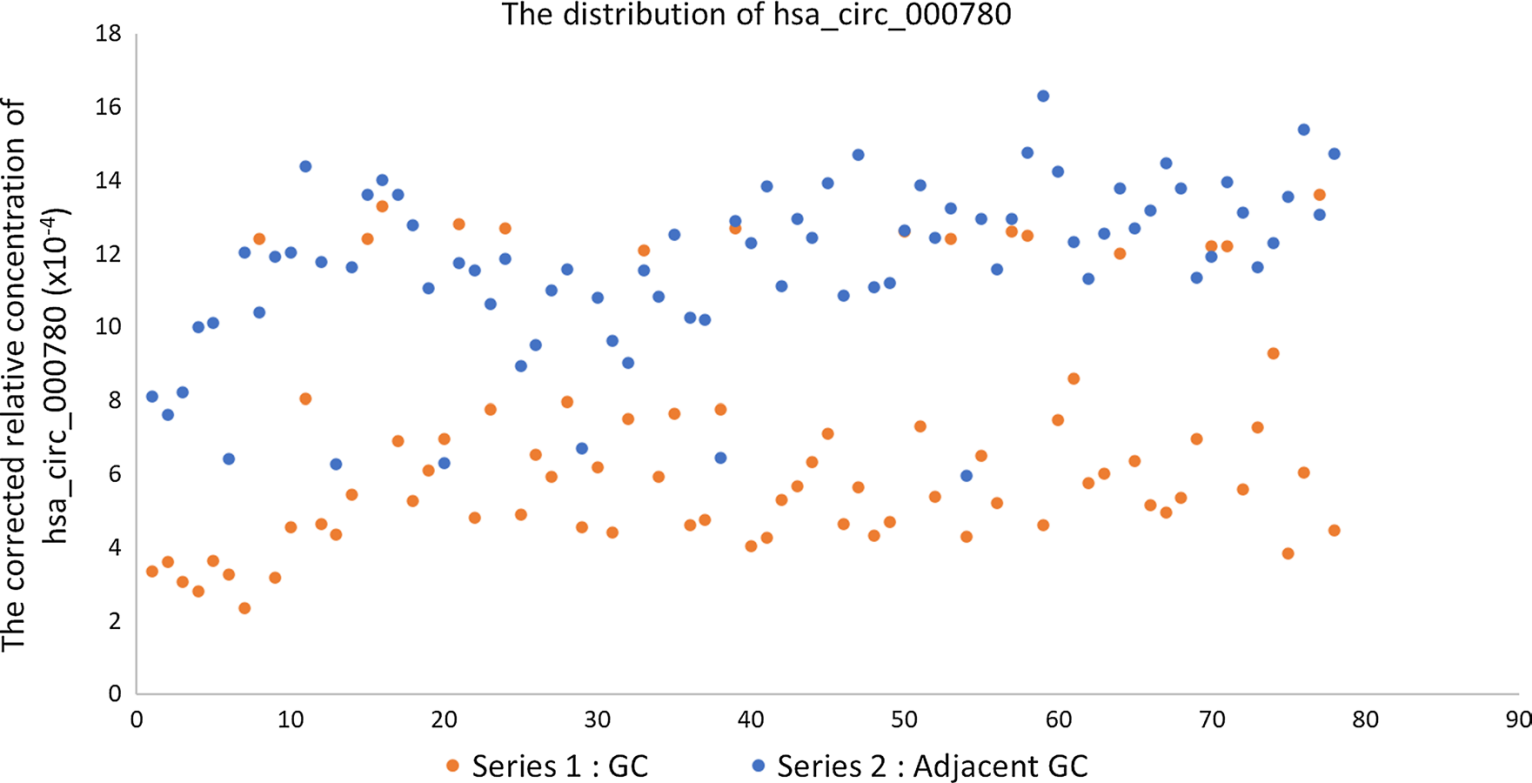
Relative level distribution of hsa_circ_000780 in GC and adjacent tissue specimens

The sample size in this study was expanded to 78 patients with GC and their matched adjacent noncancerous tissues to verify the accuracy of the above microarray and qRT–PCR data. The expression levels of hsa_circ_000780 in these tissues were measured by qRT–PCR. The relative expression levels of hsa_circ_000780 in GC and matched adjacent noncancerous tissue samples were 6.87 × 10^–4^ ± 3.12 × 10^–4^ and 11.67 × 10^–4^ ± 2.29 × 10^–4^, respectively (*P* < 0.001). The distribution of hsa_circ_000780 is shown in Fig. 2. Taking the mean value of hsa_circ_000780 expression in paracancerous tissues as the critical value for GC diagnosis, hsa_circ_000780 expression was considered to be low in 80.77% (63/78) of GC specimens, versus only 7.69% (6/78) in the paracancerous group.

**Fig. 3:**
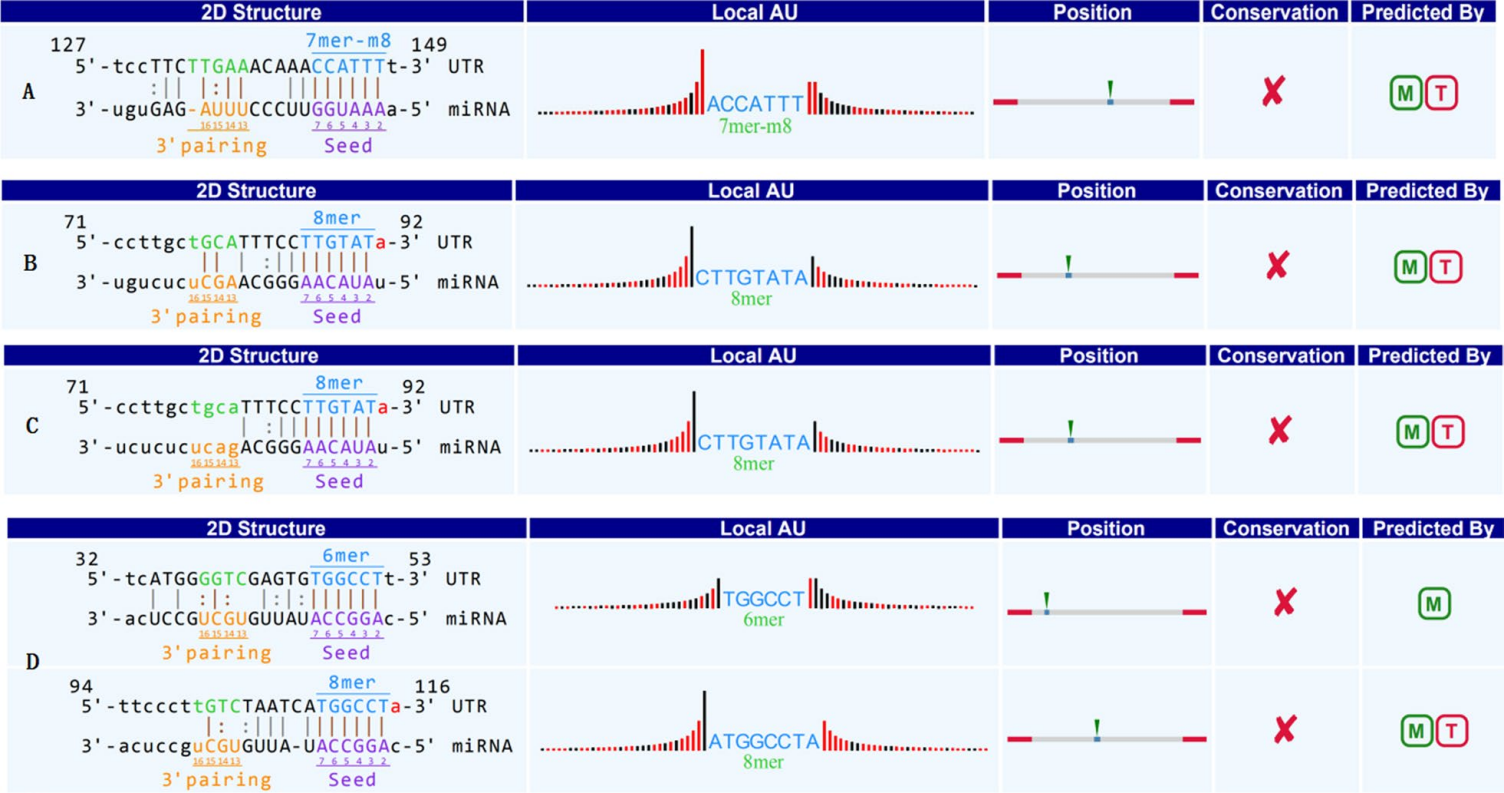
Bioinformatics analysis predicting hsa_circ_000780 interaction with **A** hsa_miR_522-3p, **B** hsa_miR_381-3p, **C** hsa_miR_300, and **D** hsa_miR_15a-3p

The PPV and NPV were 91.3% and 82.76%, respectively. Moreover, bioinformatics analysis predicted that hsa_circ_000780 could interact with hsa_miRNA_522-3p, hsa_miRNA_381-3p, hsa_miRNA_300, and hsa_miRNA_15a-3p (Fig. 3). Subsequently, correlations between hsa_circ_000780 expression and clinicopathological characteristics were analyzed. As shown in Table 4, hsa_circ_000780 expression levels in GC tissue samples were significantly correlated with tumor size (*P* = 0.020), tumor stage (*P* = 0.001), T stage (*P* = 0.029), venous invasion (*P* = 0.042), carcinoembryonic antigen (CEA) levels (*P* = 0.001), and carbohydrate antigen 19-9 (CA19-9) levels (*P* = 0.001). However, they were not significantly associated with other clinicopathological factors such as sex, age, tumor location, pathological diagnosis, lymphatic metastasis, distal metastasis, and cell differentiation (*P* > 0.05).

**Table 4:** Associations of hsa_circ_000780 levels with clinicopathological characteristics in GC

Characteristics	No. of patients (*n* = 78, %)	Expression levels of has_circ_000780 (Mean ± SD, × 10^–4^)	*P* value
Age (year)			
≥ 60	45 (57.7)	5.97 ± 1.65	1.000
< 60	33 (42.3)	5.97 ± 2.12	
Sex			
Male	40 (51.3)	6.32 ± 3.09	0.112
Female	38 (48.7)	7.45 ± 3.09	
Tumor location			
Sinuses ventriculi	39 (50.0)	6.20 ± 3.29	0.418
Cardia	17 (21.8)	7.80 ± 3.04	
Corpora ventriculi	13 (16.7)	6.90 ± 2.57	
Others	9 (11.5)	7.98 ± 2.96	
**Diameter (cm)**			
≥ 5	38 (48.7)	6.03 ± 3.16	0.020
< 5	40 (51.3)	7.67 ± 2.91	
Differentiation			
Well	9 (11.5)	7.29 ± 2.55	0.303
Moderate	36 (46.2)	7.38 ± 3.04	
Poor	32 (41.3)	6.20 ± 3.20	
**Stage**			
Early	21 (26.9)	5.08 ± 2.13	0.001
Advanced	57 (73.1)	7.53 ± 3.19	
Pathologic diagnosis			
Signet ring cell cancer	11 (14.1)	5.21 ± 2.80	0.055
Adenocarcinoma	67 (85.9)	7.14 ± 3.11	
**T stage**			
T1 and T2	25 (32.1)	5.78 ± 2.84	0.029
T3 and T4	53 (67.9)	7.38 ± 3.14	
Lymphatic metastasis			
N0	28 (56.0)	6.63 ± 3.44	0.625
N1-2	50 (44.0)	7.01 ± 2.96	
Distal metastasis			
M0	68 (87.2)	5.81 ± 3.74	0.345
M1	10 (12.8)	7.03 ± 3.02	
**Venous invasion**			
Absent	41 (51.3)	6.19 ± 3.20	0.042
Present	37 (51.3)	7.62 ± 2.89	
**Carcinoembryonic antigen**			
Positive	25 (52.6)	5.22 ± 2.63	0.001
Negative	53 (47.1)	7.65 ± 3.05	
**CA19-9 (Tissue)**			
Positive	21 (26.9)	4.67 ± 2.14	0.001
Negative	57 (73.1)	7.68 ± 3.05	

Bold values: *P* < 0.05

### Amounts of hsa_circ_000780 in gastric juice specimens

**Fig. 4:**
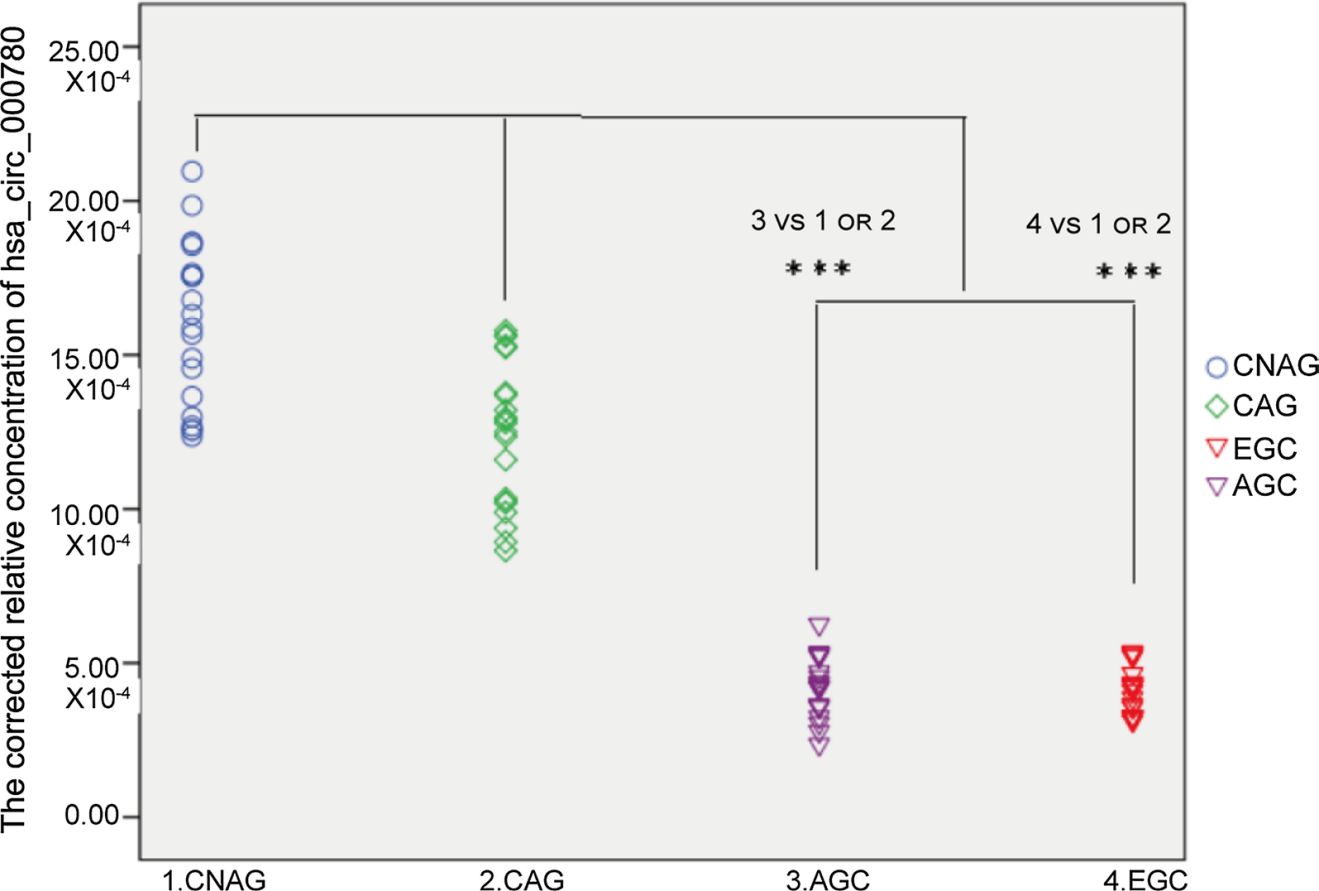
Relative expression levels of hsa_circ_000780 in gastric juice samples. Hsa_circ_000780 levels in the gastric juice at various stages of GC, including CNAG (*n* = 30), CAG (*n* = 30), EGC (*n* = 21), and AGC (*n* = 57), were detected by qRT-PCR (***P < 0.001)

Next, hsa_circ_000780 levels in gastric fluid samples from 30 patients with CNAG, 30 with CAG, 21 with EGC, and 57 with AGC were assessed by qRT–PCR. The values for the CNAG, CAG, EGC, and AGC groups were (15.63 ± 2.44) × 10^–4^, (12.59 ± 2.13) × 10^–4^, (4.28 ± 0.98) × 10^–4^, and (4.39 ± 1.15) × 10^–4^, respectively (Fig. 4). The expression levels of hsa_circ_000780 significantly differed in the CNAG and CAG groups compared with the EGC and AGC groups (*P* < 0.001). The hsa_circ_000780 levels were significantly decreased in the gastric fluid of the GC group. No significant difference in hsa_circ_000780 levels was found between the AGC and EGC groups (*P* > 0.05) or between the CNAG and CAG groups (*P* > 0.05). Taking the mean value of hsa_circ_000780 expression in CNAG, CAG and GC *gastric juice specimens* as the critical level for GC diagnosis, hsa_circ_000780 expression was considered to be low in 100% (78/78) of GC *juice specimens*, versus 0% (0/60) in the gastritis group. The PPV and NPV were both 100%.

## Discussion

Several studies have demonstrated the involvement of circRNAs in the proliferation, apoptosis, invasion, and metastasis of human tumors [21, 26, 32]. Huang et al. [33] reported 16 upregulated and 84 downregulated circRNAs in GC. Of these, only hsa_circ_0000026 was downregulated by a fold change of 2.8 in GC as detected by qRT-PCR, and this difference was significant. Dang et al. [34] examined the expression profiles of five pairs of GC and matched non-GC tissues, and found 713 differentially expressed circRNAs in GC, including 191 and 522 upregulated and downregulated, respectively. Shen et al. [32] performed circRNA microarray analysis and stated that 347 upregulated and 603 downregulated circRNAs were observed in GC compared with normal gastric tissue. Of 20 randomly selected circRNAs, 10 were confirmed to have differential expression. The circRNA microarray results in the present study revealed a new circRNA expression profile in human GC, and the differentially expressed circRNAs detected above showed a significant difference compared with those reported in other studies [26, 34]. This study showed that 445 circRNAs were significantly dysregulated in GC. Of these, 15.51% were upregulated and 84.49% were downregulated. The trend of downregulated circRNA expression observed in this study was similar to that reported in other studies. However, through reverse verification in GC tissues and paired adjacent tissues, the expression levels of has_circ_000780, has_circ_047478, has_ circ_404798 and has_circ_104293 were significantly different. A literature search of the PubMed database (https://www.ncbi.nlm.nih.gov/PubMed/) until November 8, 2019 did not retrieve these circRNAs in GC. These results suggested the genetic heterogeneity of GC. In addition, most differentially expressed circRNAs in this study were found on human chr1, chr3, chr4, chr6, and chr11. Shao et al. [26] observed that the differentially expressed circRNAs were mainly transcribed from chr1 and chr3, suggesting that despite the great heterogeneity in the genetic mechanism of GC, there are overlaps in the expression of circRNAs. This finding may provide a direction for further investigation of GC pathogenesis and diagnostic targets.

The circRNA expression profiles in GC further confirmed that circRNAs are closely associated with GC. However, only few circRNAs have been shown to regulate carcinogenesis in GC [35–38]. In the present study, hsa_circ_000780 was selected as a target circRNA to validate the accuracy of microarray results. The results revealed that hsa_circ_000780 was significantly downregulated in 80.77% of GC tissue samples. Bioinformatics analysis predicted that hsa_circ_000780 could interact with hsa_miRNA_522-3p, hsa_miRNA_381-3p, hsa_miRNA_300, and hsa_miRNA_15a-3p. MicroRNAs (miRNAs) are a class of small noncoding RNAs of 20–22 nucleotides in length, which play an important role in regulating gene expression by directly binding to the 3’-untranslated regions (3’-UTRs) of target mRNAs [39]. It has been demonstrated in a number of studies that miRNAs are among the pivotal factors in many biological processes, including cell differentiation, cell proliferation, apoptosis, and energy metabolism [40]. Moreover, recent studies have revealed that miRNAs play a dual role in oncology either by enhancing carcinogenesis through inhibiting tumor suppressors or acting as tumor suppressors to downregulate oncogenes. MiR-522-3p up-regulation negatively regulates BLM, with upregulation of c-myc, CDK2 and cyclin E, thereby promoting the proliferation of human CRC cells [41]. MiR-522-3p is an oncogene in glioblastoma by targeting SFRP2 through the Wnt/β-catenin pathway [42]. The miR-381-3p/RAB2A axis induces cell proliferation and inhibits cell apoptosis in bladder cancer [43]. MiR-381-3p targeted and suppressed the NASP gene, and reduced viability, migration, invasion and EMT in HNSCC cells [44]. MiR-300/FA2H affects gastric cancer cell proliferation and apoptosis. The OIP5-AS1/miR-300/YY1 feedback loop facilitates cell growth in HCC by activating the WNT pathway [45, 46]. MiR-15a-3p may contribute to adenoma-to-carcinoma progression. MiR-15a-3p and miR-16–1-3p negatively regulate Twist1 to repress gastric cancer cell invasion and metastasis [47, 48]. The literature suggests that circular RNAs can regulate the occurrence, growth and metastasis of tumors through a variety of signaling pathways. Additionally, hsa_circ_000780 expression levels in GC were significantly associated with tumor size, stage, degree of invasion, and CEA and CA19-9 expression levels, suggesting that hsa_circ_000780 has the potential to predict clinical prognosis. The gastric juice is a good sample for use in the diagnosis of gastric diseases. In the present study, we further evaluated the expression of hsa_circ_000780 in gastric juice samples from patients with CNAG, CAG, EGC, and AGC. Although hsa_circ_000780 levels in the gastric juice of GC patients were obviously decreased, there was no significant difference between the EGC and AGC groups. This finding indicates that hsa_circ_000780 could be detected in the gastric juice, and has the potential for use as a biomarker for early GC screening.

## Conclusions

In conclusion, the present study found a new expression profile of circRNAs in GC. Among the circRNAs detected, hsa_circ_000780 was significantly downregulated in GC, suggesting that it might be involved in the occurrence of GC. The level of this circRNA was related to some clinicopathological characteristics of GC patients. However, its role and mechanism in the occurrence of GC must be further investigated. Interestingly, hsa_circ_000780 could be detected in the gastric juice in early GC, with a significant difference compared with the control group. Therefore, this circRNA has the potential to be used as a novel biomarker for the screening of early GC. However, the sample size of the current study was not large enough, and the research conclusions still need to be further verified.

## Data Availability

The datasets generated and/or analyzed during the current study are available in the [GEO, GSE184882] repository, [https://www.ncbi.nlm.nih.gov/gds/?term=GSE184882/].

## Abbreviations

circRNA: Circular RNA
GC: Gastric cancer
PCR: Polymerase chain reaction
EGC: Early gastric cancer
AGC: Advanced gastric cancer
NCCN: National Comprehensive Cancer Network
CNAG: Chronic nonatrophic gastritis
RT: Reverse Transcription
FC: Fold change
qRT-PCR: Quantitative reverse transcription–polymerase chain reaction
